# Who Are the High-Cost Users? A Method for Person-Centred Attribution of Health Care Spending

**DOI:** 10.1371/journal.pone.0149179

**Published:** 2016-03-03

**Authors:** Sara J. T. Guilcher, Susan E. Bronskill, Jun Guan, Walter P. Wodchis

**Affiliations:** 1 Leslie Dan Faculty of Pharmacy, University of Toronto, Toronto, Canada; 2 Health System Performance Research Network, Toronto, Ontario; 3 Centre for Research of Inner City Health, Li Ka Shing Knowledge Institute, St. Michael’s Hospital, Toronto, Canada; 4 Institute for Clinical Evaluative Sciences, Toronto, Canada; 5 Institute of Health, Policy, Management and Evaluation, University of Toronto, Toronto, Canada; 6 Toronto Rehabilitation Institute-University Health Network, Toronto, Ontario; University Hospital Oldenburg, GERMANY

## Abstract

**Objective:**

To develop person-centered episodes of care (PCE) for community-dwelling individuals in the top fifth percentile of Ontario health care expenditures in order to: (1) describe the main clinical groupings for spending; and (2) identify patterns of spending by health sector (e.g. acute care, home care, physician billings) within and across PCE.

**Data sources:**

Data were drawn from population-based administrative databases for all publicly funded health care in Ontario, Canada in 2010/11.

**Study design:**

This study is a retrospective cohort study.

**Data collection/extraction methods:**

A total of 587,982 community-dwelling individuals were identified among those accounting for the top 5% of provincial health care expenditures between April 1, 2010 and March 31, 2011. PCE were defined as starting with an acute care admission and persisting through subsequent care settings and providers until individuals were without health system contact for 30 days. PCE were classified according to the clinical grouping for the initial admission. PCE and non-PCE costs were calculated and compared to provide a comprehensive measurement of total health system costs for the year.

**Principal findings:**

Among this community cohort, 697,059 PCE accounted for nearly 70% ($11,815.3 million (CAD)) of total annual publicly-funded expenditures on high-cost community-dwelling individuals. The most common clinical groupings to start a PCE were Acute Planned Surgical (35.2%), Acute Unplanned Medical (21.0%) and Post-Admission Events (10.8%). Median PCE costs ranged from $3,865 (IQR = $1,712-$10,919) for Acute Planned Surgical to $20,687 ($12,207-$39,579) for Post-Admission Events. Inpatient acute ($8,194.5 million) and inpatient rehabilitation ($434.6 million) health sectors accounted for the largest proportions of allocated PCE spending over the year.

**Conclusions:**

Our study provides a novel methodological approach to categorize high-cost health system users into meaningful person-centered episodes. This approach helps to explain how costs are attributable within individuals across sectors and has applications in episode-based payment formulas and quality monitoring.

## Introduction

Improving health system value and efficiency are top policy issues around the globe [1, 2]. Moreover, there is a widely accepted recognition that growth patterns of total health system costs are unsustainable, particularly with demographic challenges of an aging population. There is a pertinent need to arrest total health care spending while also providing person-centred and value-based care [2, 3]. It has been well reported across numerous jurisdictions and over time that health care spending is skewed, with a minority of the population driving health care costs [4–9]. Previous work has shown 1% of the population in the United States accounted for 20% of the total system costs [6, 9] and the top 5% accounted for approximately 50% of total expenditures. Similar findings have been reported in the United Kingdom [10], Australia [7], and Canada [5, 11–15].

Healthcare payers have traditionally focused on payments to providers within specific sectors such as acute, physician, pharmacy and other providers. Recently, there are renewed efforts to draw attention away from sector specific costs and focus on the characteristics of individuals who are the drivers of healthcare spending, such as profiling by patient, physician, and health care market characteristics [16–19]. Understanding costs from the perspective of individuals who consume a large proportion of cost is important in order to inform and target improvements in efficiency, effectiveness and enhanced quality of care for targeted groups [20, 21]. Conway and colleagues made advancements in this area by categorizing health expenditures in the United States into patient-centred categories [22]. In this study, Conway and colleagues identified that the majority of health spending was related to chronic conditions followed by acute illness, trauma/poisoning, dental, preventive health and pregnancy [22]. While Conway’s work advances the field in understanding categories of high-cost users [22], the analysis did not track individual patients over time through interactions with different health care sectors across the continuum of care. Moreover, there still remains a gap in understanding the characteristics of persons with high needs and the underlying factors that may be driving costs along the continuum of care.

Importantly, better understanding of patient profiles enables interventions and policies to be more strategically targeted [22]. Given rising health care costs, it is becoming increasingly important to provide efficient value-based health care that is needs driven to specific populations and individual episodes across the continuum of care [2, 3, 23]. Targeting improvement interventions to modifiable factors among high-cost populations is an obvious approach but necessitates characterizing high-cost populations and matching interventions to applicable populations. Expanding cost methodology and tracking care through episodes across the continuum of care and over time supports such intervention implementation and policy planning [22, 24], particularly for persons in the top 5% of health care expenditures.

The purpose of this study is to understand the main clinical groupings for which individuals have intensive interactions with the health care system that incur high-costs, and to examine the utility of person-centred episodes of care (PCE) amongst these individuals. Specifically, we developed and used PCE for individuals in the top fifth percentile of health spending to describe the main causes for expenditures; and (2) to describe costs related to specific episodes overall and by health sector (e.g., emergency department, hospitalizations, home care, physician billings).

## Materials and Methods

### Design

This is a retrospective cohort study.

### Setting

The province of Ontario is located in central Canada and is the most populous province with over 13 million residents, representing 40% of the Canadian population [25]. Ontario has a universal public health care system, the Ontario Health Insurance Plan (OHIP), which is paid for by the Ontario Ministry of Health and Long-Term Care (MOHLTC) from general taxation revenues. The MOHLTC pays for all medically necessary physician and hospital-based care (free at the point of care), as well as home care and long-term care services. For persons aged 65 or over, those supported by provincial social assistance payments, and/or those with relatively high drug costs, the MOHLTC provides pharmaceutical coverage subject to an income tested nominal dispensing fee co-payment. Long-term care residents pay for the cost of room and board based on a ministry regulated co-payment structure [26], otherwise the public system provides cost-free care at the point of service. Residents pay privately for dental care, eye care, outpatient rehabilitation (e.g., chiropractic, physiotherapy, naturopathic), and other services.

### Data Sources

All publicly funded health care encounters are captured in health administrative databases collected and stored by the Canadian Institute for Health Information (CIHI) and the MOHLTC. The cohort for this study was derived from the Registered Persons Database, which contains basic demographic and vital statistics information on all persons who are eligible for provincial health insurance. Data from sectors along the continuum of publicly funded care were linked to the base cohort over time and included: hospital records from acute care (inpatient acute, designated inpatient mental health care, and same day surgery); emergency department; inpatient rehabilitation; inpatient complex-continuing care; residential long-term care; physician billings; and outpatient drug prescriptions for eligible individuals (aged 65 years or over, supported by provincial social assistance payments, and/or those with relatively high drug costs). Ontario’s health administrative data has been shown to be both valid and reliable [27]. These data sources were linked to individuals using unique encoded identifiers and analyzed at the Institute for Clinical Evaluative Sciences (ICES, Toronto, Ontario). Patient records were anonymized and de-identified prior to analysis. Approval to complete this study was granted by the Sunnybrook Health Sciences Centre Research Ethics Board.

### Population

We identified more than 13 million individuals with Ontario health insurance coverage as of April 1, 2010 (or newborns/immigrants between April 1, 2010 to March 31, 2011), who had at least one interaction with the Ontario health care system within the prior 5 years, and were alive at the index date of April 1, 2010.

### Health care costs

Health care costs were estimated on publicly-funded health care coverage. Cumulative publicly funded health care system costs were calculated using an established costing methodology at ICES, which allocates costs to various health care system encounters at the individual level over time [14, 15, 26]. Resource utilization intensity within Canadian acute hospitals is measured with Resource Intensity Weights (RIWs). RIWs are assigned to each acute hospital encounter and reflect the average amount of hospital resources (e.g., administration, staff, supplies, drugs, technology, and equipment). RIWs are multiplied by a cost per weighted case to estimate the total cost for a specific encounter within acute care admission. Ontario has adopted CIHI’s RIW methodology for acute care, emergency department, same day surgery, inpatient rehabilitation, inpatient complex-continuing care, residential long-term care, and designated inpatient mental health care. Admissions to inpatient rehabilitation, inpatient mental health, and inpatient complex-continuing care have appropriate weights applied for the specific type of care setting. Cost for long-term care is measured by fixed per diem costs based on government payment rates. Physician costs are captured by claims submitted to the OHIP directly by physicians in private practice. Primary care physicians are also remunerated with capitation payments based on the payment rate and the particular model of primary care for each patient’s physician in each month of the study period. Drug costs capture all costs for prescription drugs dispensed in outpatient pharmacies to individuals eligible for publicly funded drug coverage (Ontario Drug Benefit program). Home care costs are allocated to individuals for each visit according to the type of service; case management and administration costs are allocated on a per-case basis. Amounts reimbursed to individuals for assistive devices are captured through the Assistive Device Program database.

**Fig 1:**
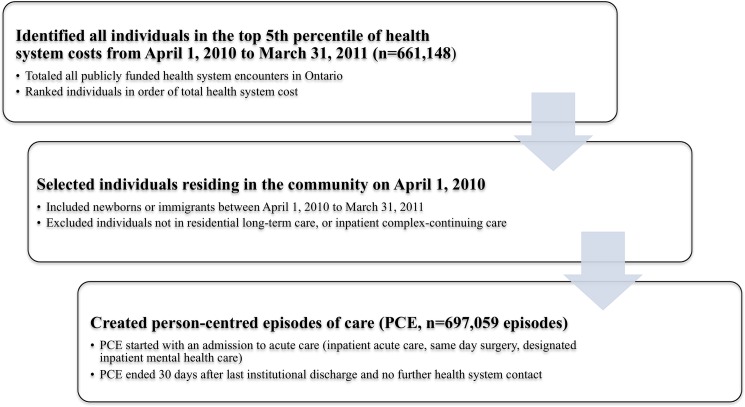
Identification of high cost health users originating in the community and creation of person-centred episodes of care (PCE), April 1, 2010 to March 31, 2011.

For the period from April 1, 2010 to March 31, 2011, we totaled costs for all publicly funded health system encounters at the individual-person level (see Fig 1). These encounters included acute care, emergency department, same day surgery, inpatient rehabilitation, inpatient complex continuing care, residential long-term care, outpatient Ontario Drug Benefit medications, physician services, home care services and assistive device program reimbursements. These costs were calculated and summed for all health sectors using the validated algorithms at ICES as described above [14, 15, 26]. Only individuals with the highest fifth percentile of expenditures (greater than $8,522 total annual costs in 2010 Canadian dollars) and who were living in the community on April 1, 2010 were included in this study population of high-cost users (n = 587,982).

### Person-centred episodes of care

In general, institutional care is a primary driver of spending among high-cost individuals [22]. Therefore, we sought to use episodes of care that began with an initial acute hospital setting (inpatient acute care, same-day surgery, emergency department, or designated inpatient mental health care) and included subsequent care until the individual had returned to the community and was stabilized for 30 days without any institutional admissions. We adopted the 30 day window as this is a common threshold for determining quality payment bundles and for examining quality of care such as readmissions [28, 29]. We refer to these individualized episodes as PCE.

### Creation of episodes

To create the PCE, we tracked all health care encounters for each individual sequentially from April 1, 2010 to March 31, 2011. PCE were determined to begin with an admission to an acute hospital setting (inpatient acute care, same-day surgery or emergency department) or a designated inpatient mental health institution. We followed individuals forward in time for 30 days from the date of discharge for this initial service use to see if there were any subsequent institutional care admissions, transfers or readmissions. If there was a subsequent institutional admission of any sort (including inpatient acute, designated inpatient mental health, emergency department, inpatient rehabilitation, and inpatient complex-continuing care or residential long-term care), the PCE continued and a new 30 day window was examined from the discharge date of this subsequent admission. The PCE ended after individuals returned to the community with no further institutional care within the 30 days window. All care in the community (home care, physician visits, pharmacy claims) within 30 days of discharge from the final institution was included in PCE costs. Individuals were eligible to have more than one episode of care within the study period. Costs for care prior to the PCE start date and/or 30 days after the PCE end date were not included in episode costs. Fig 1 illustrates the creation of the cohort and the relevant time windows for the PCE.

### Clinical classification of episodes

For each PCE, we classified the main cause for admission based on the most responsible clinical diagnosis (MRD, with exceptions for some categories indicated below) and type of admission (medical or surgical and urgent or planned/elective) during the initial hospitalization (see S1 Fig for more details and exceptions). Guided by Conway’s previous work [22], the following hierarchy of mutually exclusive person-centered categories were created:

(1) Pregnancy; (2) Low Birth Weight, Other Perinatal and Congenital Conditions; (3) Post-Admission Events (irrespective of the MRD); (4) Trauma, Accidents, Injuries and Poisonings (irrespective of the MRD); (5) Mental Illness and Addictions; (6) Ambulatory Care Sensitive Conditions; (7) Cancer; (8) Acute Planned Surgical; (9) Acute Planned Medical; (10) Acute Unplanned Surgical; (11) Acute Unplanned Medical; (12) Other Causes. The exception to the MRD-rule was assignment to the Post-Admission Event category which was made before assignment to other categories if the patient had any post-admission diagnosis (e.g., complications of devices, procedures) coded on their initial acute admission record (Post-Admission Events were not assessed for Pregnancy and Low Birth Weight, Other Perinatal and Congenital Conditions as they were identified prior to this category in the hierarchy). Trauma, Accidents, Injuries and Poisonings included any of the diagnosis codes that were selected for this category (see S1 Fig for more details). Otherwise individuals were categorized based on the MRD or whether they were admitted for surgical procedures. We considered additional sub-classifications for high frequency diagnoses within medical and surgical categories. Cancer was the only diagnosis group with sufficient numbers (>2%) to warrant a disease-specific category. Admissions to inpatient mental health facilities in Ontario were classified in the Mental Illness and Addictions category. Ambulatory visits for outpatient oncology treatments were allocated to the Cancer category during the episode of care.

### Expenditures for episodes

Total expenditures were calculated for each PCE based on all utilization from the index admission date until the end of the episode. All costs from all sectors within the episode (from initial admission through to 30 day follow up period) were attributed to each PCE. Episode-specific costs were compared to total costs for each individual during the study period. All costs are reported in 2010 Canadian dollars.

### Characteristics of high-cost users

For each individual, we described age, sex, rurality, main primary care setting, usual provider of ambulatory care index, morbidity burden, number of different drugs for those eligible to receive coverage under the Ontario Drug Benefit plan, history of palliative care service use, and death during the study period. Rurality was determined by the Rurality Index of Ontario (RIO), which is a scaled index based on population factors and distance (ranges 0–100), and communities where higher values (cut point ≥ 40) are considered rural [30]. In Ontario, primary care services are organized around different models of care (Family Health Teams, Capitation and Fee for Service) and these models have been shown to serve different patient populations and outcomes [31, 32]. The main primary care setting, as of the study start date (April 1, 2010), assessed whether the patient was rostered to a team-based care model (Family Health Team, which provides care most closely approximating a patient-centred medical home), or whether their physician was paid primarily on a capitation or fee-for-service basis [31, 32].

The Ambulatory Usual Provider of Care (UPC) index determines the proportion of all ambulatory care visits in the two years prior to the study year to the most highly visited provider (ambulatory visits were defined as a subset of physician visits by location in the OHIP database, e.g., office, home, phone). A two-year look back was used to determine the Ambulatory UPC index, and calculated for individuals with at least three physician visits in the two year period. A threshold of 75% of visits with the same physician was used to indicate high continuity [33].

In order to determine morbidity burden, we used the John Hopkins Aggregated Diagnosis Groups (ADGs) scores, which are calculated using the Johns Hopkins University Adjusted Clinical Group (ACG) system [34]. In using ADGs, individuals were assigned to one of 32 different groups based on their utilization of physician and hospital services over the two years prior to index date. The total number of ADGs identified for each individual provides a measure of the number of co-occurring morbidities based on grouping diagnosis codes by severity and likelihood of persistence [34]. Applying ADGs with administrative health data for an Ontario population has been previously validated at ICES [35].

Using the Ontario Drugs Benefit Claims database, the number of different drugs dispensed to eligible persons was identified for the year prior to the study index date (April 1, 2009 to March 31, 2010). Palliative care was identified based on any physician billing or treatment in an acute hospital for palliative care during the study period. Deaths were captured by a recorded death between April 1st, 2010 and March 31st, 2011 from the Ontario Registrar General Death database or the Registered Persons Database.

### Statistical analyses

While the focus of this research was to examine costs associated with PCE, descriptive statistics were used to summarize patterns in demographic and clinical characteristics among high-cost users and to outline the frequency of the main causes for the PCE. Distributions of expenditures within and across episodes of care were characterized by means (standard deviations) and medians (interquartile ranges). The proportion of total costs for attributed to PCE was examined.

## Results

### Characteristics of high-cost users

Table 1 summarizes the characteristics of the top fifth percentile of high-cost health system users residing in the community at index date (n = 587,982). Slightly less than half of the cohort was younger than 65 years of age (47.9%). There were slightly more females (53.5%), and the majority of high-cost users lived in an urban environment (89.5%). The majority of these individuals received primary care in a Fee for Service model (59.0%) while 21.4% were primarily in a Capitation model and 17.9% were enrolled in a Family Health Team. Additionally, the majority of the cohort had a low continuity of ambulatory care, as 77.7% had less than 0.75 on the UPC index.

**Table 1:** Socio-demographic and clinical characteristics of individuals originating in the community among high-cost health system users, April 1, 2010.

Characteristics	Category	Number of Individuals with Community Origin (n = 587,982)
**Age Group**	0 to 17	28,070 (4.8%)
	18 to 44	89,232 (15.2%)
	45 to 64	164,182 (27.9%)
	65–84	239,453 (40.7%)
	85+	67,045 (11.4%)
**Sex**	Female	314,550 (53.5%)
	Male	273,432 (46.5%)
**Rurality Index of Ontario**	Urban (0–39)	526,441 (89.5%)
	Rural (≥40)	55,785 (9.5%)
	Missing	5756 (1.0%)
**Primary Care**	Family Health Team	111,375 (17.9%)
	Primarily Fee for Service	346,908 (59.0%)
	Primarily Capitation	125,935(21.4%)
	Other	9,754 (1.6%)
**Usual Provider of Ambulatory**	No health system contact	33,640 (5.7%)
**Care Index**	Low (<0.75)	456,845 (77.7%)
	High (≥0.75)	97,497 (16.6%)
**Morbidity Burden^a^**	No health system contact	14,197 (2.4%)
	No chronic conditions (0 ADGs)	2,815 (0.5%)
	Few conditions (1–7 ADGs)	227,298 (38.7%)
	Many conditions (≥8 ADGs)	343,672 (58.5%)
**Eligible for Ontario Drug Benefit Coverage in Year Prior**	Not eligible	281,484 (47.9%)
	**Eligible**	306,498 (52.1%)
**# of Different Drug Therapies Dispensed in Year Prior for Persons Eligible**	0	8,239 (2.7%)
	1 to 5	54,573 (17.8%)
	6 to 9	76,085 (24.8%)
	10 to 19	135,226 (44.1%)
	20 +	32,375 (10.6%)
**Received Palliative Care During the Year**	Yes	68,996 (11.7%)
**Died During the Year**	Yes	52,999 (9.0%)

^a^ADG = Aggregated Diagnosis Group.

Individuals had significant morbidity burden, as 58.5% of the cohort had eight or more distinct comorbid conditions as per the ADGs, and less than 1% of the cases were identified with no pre-existing comorbid condition. Further, a substantial proportion of individuals who received Ontario Drug Benefit were prescribed multiple concurrent drug therapies, as 54.7% were prescribed 10+ different drugs. A total of 68,996 persons (11.7%) received palliative care during the year and 9.0% of individuals died. One-year expenditures for health services for the top fifth percentile of high-cost users originating in the community totaled $17,203 million.

### Clinical grouping of person-centred episodes of care

A total of 697,059 PCE were identified among individuals in the cohort, and 82.6% of individuals had at least one PCE. Table 2 shows the main clinical groupings associated with the initiation of a PCE and the distribution of costs across groupings. Acute Planned Surgical (35.2%, n = 245,329 episodes), Acute Unplanned Medical (21.0%, 146,079 episodes), and Post-Admission Events (10.8%, n = 75,126 episodes) were the most common PCE clinical groupings. Post-Admission Events accounted for the largest proportion of PCE-related expenditures (23.1%; median cost per episode $20,687; IQR = $12,207-$39,579), followed by Acute Unplanned Medical (21.1%; median cost per episode $9,505, IQR = $6,373-$17,981) and then Acute Planned Surgical (16.0%; median cost per episode $3,865; IQR = $1,712-$10,919). It is also noteworthy that, due to high individual costs, Trauma, Accidents, Injuries and Poisonings, and Mental Illness and Addictions accounted for 10.1% and 10.8% of overall costs associated with PCE but only 5.9% and 3.9% of total PCE.

**Table 2:** Distribution of person-centred episodes of care (PCE) and related costs among high-cost health system users originating in the community by clinical grouping, April 1, 2010 to March 31, 2011.

Clinical Groupings for PCE, Ordered Based on Total Costs*	Number of PCE (%)	Average Cost per PCE $ (SD)	Median Cost per PCE $ (IQR)	Total Costs for PCE
				$Millions (%)
***All PCE***	**697,059 (100)**	**30,961 (38,069)**	**18,251 (12,160–33,978)**	**11,815.3 (100)**
***Post-Admission Events***	75,126 (10.8)	36,303 (52,279)	20,687 (12,207–39,579)	2,727.3 (23.1)
***Acute Unplanned Medical***	146,079 (21.0)	17,057 (23,515)	9,505 (6,373–17,981)	2,491.6 (21.1)
***Acute Planned Surgical***	245,329 (35.2)	7,717 (12,291)	3,865 (1,712–10,919)	1,893.3 (16.0)
***Mental Illness & Addictions***	41,327 (5.9)	30,948 (44,262)	17,224 (10,325–32,126)	1,279.0 (10.8)
***Trauma*, *Accidents*, *Injuries*, *Poisonings***	44,326 (6.4)	26,971 (34,807)	15,753 (8,947–31,731)	1,195.5 (10.1)
***Cancer***	51,825 (7.4)	13,521 (18,500)	9,073 (4,113–15,298)	700.7 (5.9)
***Acute Unplanned Surgical***	28,574 (4.1)	18,676 (22,155)	13,647 (8,900–19,356)	533.6 (4.5)
***Ambulatory Care Sensitive Conditions***	26,911 (3.9)	14,719 (20,987)	8,416 (6,022–14,750)	396.1 (3.4)
***Low Birth Weight***	10,714 (1.5)	27,491 (42,026)	15,492 (9,863–29,135)	294.5 (2.5)
***Pregnancy***	18,095 (2.6)	8,095 (6,552)	7,255 (5,243–8,974)	146.5 (1.2)
***Acute Planned Medical***	7,783 (1.1)	17,281 (25,539)	9,679 (5,759–18,839)	134.5 (1.1)
***Other Causes***	970 (0.1)	23,283 (33,368)	11,965 (4,912–26,509)	22.6 (0.2)

*Clinical groupings for PCE were based on the first acute encounter that started the PCE.

PCE were determined to begin with an admission to an acute hospital setting (inpatient acute care, same-day surgery or emergency department) or inpatient mental health institutions.

We further investigated the most common specific conditions associated with each category of spending (full detail on the top 10 health conditions that comprise the clinical groupings can be found in S2 Fig). Interestingly, for the Post-Admission Events grouping, several of the conditions were related to complications with procedures (11.3%, n = 8,459 events) and complications with orthopedic devices (4.9%, n = 3,692 events).

### Health service use and related costs during person-centred episodes of care

Table 3 shows the distribution of health services used during the PCE. Close to seventy-two percent of PCE included at least one inpatient acute care admission; 46.6% involved an emergency department visit; almost all included contact with a specialist physician; and 79.2% had at least one visit to a general practitioner. Within the PCE, inpatient acute care comprised the largest amount of total cost ($6,882.5 million), followed by physician services ($1,495.4 million) and designated inpatient mental health care ($974.2 million).

**Table 3:** Distribution of health services used within person-centred episodes of care (PCE) and related costs among high-cost health system users originating in the community, April 1, 2010 to March 31, 2011.

Type of Health Service in PCE	Distribution of Health Services within PCE	Distribution of Costs across PCE
**Inpatient acute care, $ total**		**$6,882,515,141**
**Number of PCE with any inpatient acute care admissions, n (%)**	**500,388 (71.8%)**	
Total number of admissions within PCE	644,523	
Mean ± SD	1.3 ± 0.7	$11,705 ± 24,547
**Same day surgery, $ total**		**$337,743,142**
**Number of PCE with any same day surgery visits, n (%)**	**233,140 (33.5%)**	
Total number of visits within PCE	277,518	
Mean ± SD	1.2 ± 1.0	$574 ± 1,610
**Emergency department, $ total**		**$251,768,375**
**Number of PCE with any emergency department visits, n (%)**	**324,559 (46.6%)**	
Total number of visits within PCE	564,691	
Mean ± SD	1.7 ± 2.7	$428 ± 785
**Inpatient mental health, $ total**		**$974,226,518**
**Number of PCE with any inpatient mental health admissions, n (%)**	**35,719 (5.1%)**	
Total number of admissions	45,346	
Mean ± SD	**1.3 ± 0.8**	$1,657± 13,090
**Inpatient rehabilitation, $ total**		**$434,607,526**
**Number of PCE with any inpatient rehabilitation admissions, n (%)**	**27,167 (3.9%)**	
Total number of admissions	29,497	
Mean ± SD	1.1 ± 0.3	$739 ± 5,196
**Inpatient complex continuing care, $ total**		**$358,279,818**
**Number of PCE with any complex continuing care admissions, n (%)**	**10,233 (1.5%)**	
Total number of admissions	12,721	
Mean ± SD	1.0 ± 0.2	$609 ± 5,782
**Residential long-term care, $ total**		**$187,939,677**
**Number of PCE with any long-term care admissions n (%)**	**12,911 (1.9%)**	
Total number of admissions	16,041	
Mean ± SD	1.1 ± 0.4	$320 ± 2,670
**Physician services, $ total**		**$1,495,380,763**
**Number of PCE with any general practitioner visits n (%)**	**551,929 (79.2%)**	
Total number of visits	3,264,887	
Mean ± SD	5.9 ± 9.7	
**Number of PCE with any specialist visits n (%)**	**689,868 (99.0%)**	
Total number of visits	11,138,798	
Mean ± SD	16.1 ± 24.8	
**Home care services, $ total**		**$405,790,482**
**Number of PCE with any home care visits n (%)**	**211,786 (30.4%)**	
Total number of visits	3,519,786	
Mean ± SD	16.6 ± 26.7	$690 ± 1,847

### Proportion of expenditures for persons originating in the community accounted for by person-centred episodes of care

Overall, 68.7% ($11,815.3 million) of the total of health service costs for persons originating in the community ($17,203 million) were accounted for by the PCE. Among the outstanding expenditures that were not allocated to PCE, there were 102,315 individuals that were not captured by the PCE, who accumulated total costs of $2,165.9 million (14%). Table 4 shows the expenditures captured within the PCE and the remaining costs by type of health service use. Through our methodology, inpatient admissions were largely allocated to PCE: inpatient hospitalizations (acute care, same day surgery and designated inpatient mental health care) were completely allocated to PCE (100% allocated; $8,194.5 million), inpatient rehabilitation (94.9% allocated; $434.6 million) and inpatient complex continuing care (72%; $358.3 million). The largest unallocated costs were related to drugs (86.2% unallocated; $1,503.2 million, CAD), outpatient oncology (85.7% unallocated; $517.7 million, CAD); outpatient dialysis care (83.7% unallocated; $638.9 million, CAD), home care (71.8% unallocated; $1,031.8 million, CAD), and physician and laboratory (39.6% unallocated; $1,078.1 million, CAD).

**Table 4:** Proportion of total one-year costs allocated to person-centred episodes of care (PCE) among high-cost health system users originating in the community, April 1, 2010 to March 31, 2011.

Type of health service	Costs Allocated to PCE	Remaining Costs $Millions	% of Total Costs Allocated
	$Millions		to PCE
**All Services**	**$11,815.3**	**$5,387.7**	**68.7%**
Emergency Department	$251.8	$192.3	56.7%
Inpatient Acute Hospitalizations*	$8,194.5	0	100.0%
Inpatient Non-acute Hospitalizations	$0.0	$5.3	0.0%
Inpatient Rehabilitation	$434.6	$23.1	94.9%
Inpatient Complex Continuing Care	$358.3	$139.5	72.0%
Residential Long-term Care	$187.9	$254.9	42.4%
Home Care Services	$406.0	$1,031.8	28.2%
Outpatient Dialysis	$124.3	$638.9	16.3%
Outpatient Oncology	$86.5	$517.7	14.3%
Physician Services and Laboratory	$1,530.2	$989.2	60.4%
Drugs	$241.2	$1,503.2	13.8%
Assistive Devices	$0.3	$2.8	9.0%

* Inpatient Acute Hospitalizations also includes Same Day Surgery and Inpatient Mental Health.

## Discussion

Building on Conway’s previous work [22], our method of developing PCE anchored on a first admission to an acute hospital setting represents an important approach to better understanding episodes of care. Firstly, by creating the PCE in this way, we were able to capture 68.7% of overall publicly funded expenditures for community-dwelling high-cost users. Secondly, while still relatively broad, our method proved to be useful in classifying events, as we had very few episodes in the Other Causes category (n = 970 episodes, 0.1%). This enhanced understanding of person-centred categories provides novel descriptive information for targeting service delivery and developing patient-focused funding models (e.g., funding that follows the patient rather than the sector) [20].

With the PCE methodology, we tried to establish meaningful categories reflecting the heterogeneity that exists amongst high-cost users. The clinical grouping categories identified from our PCE were most commonly Planned Surgical, Unplanned Medical and Post-Acute Admission Events. While the median cost per episode related to Planned Surgical Admissions was relatively low $3,865 (IQR = $1,712-$10,919), the high volume of surgeries (n = 245,329) contributed to the high overall costs for this category. Conversely, Post-Acute Admission Events had a lower volume of events (n = 75,126) but had a much high median cost per episode ($20,687; IQR = $12,207-$39,579). A large proportion of these Post-Acute Admission Events were related to post-orthopedic surgical complications.

The identification of PCE that are lower in volume, but have higher median cost per episode is also pertinent for health system stakeholders in order to contain potential increasing costs. Our results showed that episodes related to Trauma, Accidents, Injuries and Poisonings, and Mental Health and Addictions had substantial median costs per PCE. Our findings reinforce the value of expanding strategies for mental health, given the high median costs per episode ($17,224, IQR = $10,325-$32,126) and the rising prevalence of mental health related diagnoses such as depression [36] and dementia [37]. Within the Trauma, Accidents, Injuries and Poisonings category, fracture of the femur was one of the top contributors to this category. With an aging population, and the risk for falls among senior [38], this is another noteworthy category where costs might increase substantially. Thus, minimizing the volume of these categories would be particularly important, as an increase in volume of these episodes would substantially increase the overall costs for these categories.

The creation of PCE for high-cost health system users based on common clinical groupings helps to address needs across the continuum of care [20]. Clearly, the needs of someone with more acute events such as Cancer, Pregnancy or Low Birth Weight, or Other Perinatal and Congenital Conditions will differ from an individual who is a high-cost user due to chronic conditions or complications from procedures. Focusing on categories that may be more modifiable and sensitive to targeted interventions would be a key next step for researchers and decision-makers. More recently, there have been some initiatives in characterizing the different type of archetypes of persons who are high users [39]. Vaillaincourt and colleagues classified the health-seeking behaviour and needs for person who are high users of the health system into four different categories- medical complexity/frailty, severe relapsing condition, convergence of medical/social behavioural issues, and diagnostic uncertainty [39]. Characterizing the needs within our PCE from the perspective of individuals, providers and system leaders would be an interesting next step in moving the high users research forward.

### Limitations

There are several limitations to this study and provide an opportunity for future refinement of our PCE methodology. While we captured a large proportion of institution costs to PCE, costs in the community beyond the 30 day episode window were not well captured. The requirement for an acute admission and cut off window of 30 days post last institutional discharge had implications for certain categories that incur high-costs such as outpatient oncology and dialysis. Only 14.3% of outpatient oncology related treatment costs were captured within the PCE, leaving $517.7 million costs unallocated. Similarly, for outpatient dialysis care we only allocated 16.3% of costs within episodes ($638.9 million unallocated outside the episodes). Refinement of the methodology to capture costs for categories that may require ongoing care beyond the 30 day window, such as cancer and renal care should be developed. If we included all cancer and renal related care to PCE beyond the 30 day window, approximately 11% of the total costs would be captured ($1,857.3 million). Drug costs were also not captured well within our episodes of care though these costs may be not be episodic in nature. Additionally, we only captured drug costs within and outside the episodes for persons who are eligible for Ontario Drug Benefits (those aged 65 or over, supported by provincial social assistance payments, and those with relatively high drug costs). With rising costs related to pharmaceutical costs in the community [40, 41], linking pharmaceutical costs to episodes is an important area for future work. This might include understanding new pharmaceutical treatments that are started during an episode of care. Further modification to the mental health and addictions category would be of value, as this category represents a heterogeneous population ranging from mood disorders to dementia. Separating dementia-related diagnoses from the mental health and addictions category would be useful to reflect the variation in care needs and targeted interventions.

## Conclusion

While these categories are relatively broad, the methods introduced here provide an approach to understand patient episodes of care and to relate these to patient characteristics and interactions across the continuum of care. These methods may be useful in setting the foundation for episode-related performance measurement and payment for high-cost patient groups. Refinement of this method has potential to facilitate service organization, care planning and payment for high-cost patients across all care providers.

## Supporting Information

S1 Fig Main clinical groupings for person-centred episodes (PCE). (PDF)

S2 Fig Ten most prevalent conditions in each of the 12 defined person-centred categories. (PDF)

## References

[1] Chisholm D, Evans DB. Improving health system efficiency as a means of moving towards universal coverage. World Health Report (2010), Background Paper, 28 [Internet]. 2010. Available: http://www.who.int/healthsystems/topics/financing/healthreport/28UCefficiency.pdf. Accessed 02 February 2016.

[2] Berwick DM, Nolan TW, Whittington J. The Triple Aim: Care, Health, And Cost. Health Aff (Millwood). 2008;27(3):759–69. 10.1377/hlthaff.27.3.759 18474969

[3] Porter ME. What is value in health care? N Engl J Med. 2010;363(26):2477–81. 10.1056/NEJMp1011024 21142528

[4] Berk ML, Monheit AC. The concentration of health expenditures: an update. Health Aff (Millwood). 1992;11(4):145–9. 10.1377/hlthaff.11.4.145 1483633

[5] Roos N, Burchill C, Carriere K. Who are the high hospital users? A Canadian case study. J Health Serv Res Policy. 2003;8(1):5–10. Epub 2003/04/10. 10.1258/13558190360468164 12683428

[6] Stanton MW, Rutherford MK. The high concentration of U.S. health care expenditures. Rockville (MD): 2005.

[7] Calver J, Bramweld KJ, Preen DB, Alexia SJ, Boldy DP, McCaul KA. High-cost users of hospital beds in Western Australia: A population-based record linkage study. Med J Aust. 2006;184(8):393–7. 16618238 10.5694/j.1326-5377.2006.tb00289.x

[8] Radcliff TA, Côté MJ, Duncan RP. The identification of high-cost patients. Hospital topics. 2005;83(3):17–24. 16294676 10.3200/HTPS.83.3.17-24

[9] Cohen S, Yu W. The Concentration and Persistence in the Level of Health Expenditures over Time: Estimates for the U.S. Population, 2008–2009. Statistical Brief #354, [Internet]. 2012 February 2, 2016. Available: http://www.meps.ahrq.gov/mepsweb/data_files/publications/st354/stat354.pdf. Accessed 02 February 2016.

[10] Billings J, Mijanovich T, Dixon J, Curry N, Wennberg D, Darin B, et al. Case Finding Algorithms for Patients at Risk of Re-Hospitalisation PARR1 and PARR2. 2006. Available: http://www.kingsfund.org.uk/sites/files/kf/PARR_Case_Finding_Algorithms_PARR1_and_PARR2_Report1.pdf. Accessed 02 February 2016.

[11] Roos NP, Shapiro E, Tate R. Does a small minority of elderly account for a majority of health care expenditures? A sixteen-year perspective. Milbank Q. 1989;67(3–4):347–69. 2517569

[12] Reid R, Evans R, Barer M, Sheps S, Kerluke K, McGrail K, et al Conspicuous consumption: characterizing high users of physician services in one Canadian province. J Health Serv Res Policy. 2003;8(4):215–24. 10.1258/135581903322403281 14596756

[13] Deber R, Lam K. Handling the High Spenders: Implications of the Distribution of Health Expenditures for Financing Health Care (online paper). APSA 2009 Toronto Meeting Paper Available: http://ssrncom/abstract=1450788 [Internet]. Accessed 02 February 2016.

[14] Wodchis WP, Austin P, Newman A, Corallo A, Henry D. The Concentration of Health Care Spending: Little Ado (yet) About Much (money) Canadian Association for Health Services and Policy Research; 2012 May 30, 2012; Montreal. Available: https://www.cahspr.ca/en/presentation/5244423937dee8014beea024. Accessed 02 Feburary 2016.

[15] Wodchis WP, Austin PC, Henry DA. A 3-year study of high-cost users of health care. Can Med Assoc J. 2016 10.1503/cmaj.150064 PMC4754179 26755672

[16] Bodenheimer T, Berry-Millett R. Follow the money—controlling expenditures by improving care for patients needing costly services. N Engl J Med. 2009;361(16):1521–3. 10.1056/NEJMp0907185 19797277

[17] Fitzpatrick T, Rosella LC, Calzavara A, Petch J, Pinto AD, Manson H, et al Looking Beyond Income and Education: Socioeconomic Status Gradients Among Future High-Cost Users of Health Care. Am J Prev Med. 2015;49(2):161–71. 10.1016/j.amepre.2015.02.018 25960393

[18] Reschovsky JD, Hadley J, Saiontz-Martinez CB, Boukus ER. Following the Money: Factors Associated with the Cost of Treating High-Cost Medicare Beneficiaries. Health Serv Res. 2011;46(4):997–1021. 10.1111/j.1475-6773.2011.01242.x 21306368 PMC3165175

[19] Lemstra M, Mackenbach J, Neudorf C, Nannapaneni U. High health care utilization and costs associated with lower socio-economic status: Results from a linked dataset. Canadian Journal of Public Health. 2009;100(3):180–3. 19507718 10.1007/BF03405536 PMC6973662

[20] Lynn J, Straube BM, Bell KM, Jencks SF, Kambic RT. Using population segmentation to provide better health care for all: the "Bridges to Health" model. Milbank Q. 2007;85(2):185–208. 17517112 10.1111/j.1468-0009.2007.00483.x PMC2690331

[21] Vaillancourt S, Shahin I, Aggarwal P, Pomedli S, Hayden L, Pus L, et al Using archetypes to design services for high users of healthcare. Healthc Pap. 2014;14(2):37–41. 25880862 10.12927/hcpap.2015.24107

[22] Conway P, Goodrich K, Machlin S, Sasse B, Cohen J. Patient-Centered Care Categorization of U.S. Health Care Expenditures. Health Serv Res. 2011;46(2):479–90. 10.1111/j.1475-6773.2010.01212.x 21091472 PMC3064915

[23] Lynn J, Straube BM, Bell KM, Jencks SF, Kambic RT. Using Population Segmentation to Provide Better Health Care for All: The “Bridges to Health” Model. Milbank Q. 2007;85(2):185–208. 10.1111/j.1468-0009.2007.00483.x 17517112 PMC2690331

[24] Bodenheimer T, Fernandez A. High and Rising Health Care Costs. Part 4: Can Costs Be Controlled While Preserving Quality? Ann Intern Med. 2005;143(1):26–31. 10.7326/0003-4819-143-1-200507050-00007 15998752

[25] Statistics Canada. Population by year, by province and territory 2013 [updated November 25, 2013]. Available: http://www.statcan.gc.ca/tables-tableaux/sum-som/l01/cst01/demo02a-eng.htm. Accessed 02 Feburary 2016.

[26] Wodchis WP, Bushmeneva K, Nikitovic M, McKillop I. Guidelines on person level cost using administrative databases in Ontario. 2013. Available: http://www.hsprn.ca/activities/papers/hsprn_case_costing_vol1_2013.pdf. Accessed 02 Feburary 2016.

[27] Juurlink D, Preyra C, Croxford R, Chong A, Austin P, Tu J, et al. Canadian Institute for Health Information Discharge Abstract Database: A Validation Study. 2006. Available from: http://www.ices.on.ca/Publications/Atlases-and-Reports/2006/Canadian-Institute-for-Health-Information. Accessed 02 Feburary 2016.

[28] Canadian Institute for Health Information. Canadian Hospital Reporting Project Technical Notes—Clinical Indicators. 2013. Available: http://www.cihi.ca/CIHI-ext-portal/pdf/internet/CHRP_TNCI_PDF_EN. Accessed 05 September 2014.

[29] Jencks SF, Williams MV, Coleman EA. Rehospitalizations among Patients in the Medicare Fee-for-Service Program. N Engl J Med. 2009;360(14):1418–28. 10.1056/NEJMsa0803563 19339721

[30] Kralij B. Measuring Rurality—RIO2008_BASIC: Methodology and Results.2008. Available: https://www.oma.org/Resources/Documents/2008RIO-FullTechnicalPaper.pdf. Accessed 06 April 2015.

[31] Glazier RH, Klein-Geltink J, Kopp A, Sibley LM. Capitation and enhanced fee-for-service models for primary care reform: a population-based evaluation. CMAJ: Canadian Medical Association Journal. 2009;180(11):E72–E81. 10.1503/cmaj.081316 19468106 PMC2683211

[32] Glazier RH, Zagorski BM, Rayner J. Comparison of Primary Care Models in Ontario by Demographics, Case Mix and Emergency Department Use, 2008/09 to 2009/10 Toronto: Institute for Clinical Evaluative Sciences, 2012 Available: http://www.ices.on.ca/Publications/Atlases-and-Reports/2012/Comparison-of-Primary-Care-Models. Accessed 02 February 2016.

[33] Jaakkimainen L, Upshur R, Klein-Geltink J, Leong A, Maaten S, Schultz SE, et al. Primary Care in Ontario: ICES Atlas.2006. Available: http://www.ices.on.ca/~/media/Files/Atlases-Reports/2006/Primary-care-in-Ontario/Fullreport.ashx. Accessed 02 February 2016.

[34] The Johns Hopkins University ACG Case-Mix Adjustment System [computer program]. 1997.

[35] Austin PC, van Walraven C, Wodchis WP, Newman A, Anderson GM. Using the Johns Hopkins Aggregated Diagnosis Groups (ADGs) to Predict Mortality in a General Adult Population Cohort in Ontario, Canada. Med Care. 2011;49(10):932–9. 10.1097/MLR.0b013e318215d5e2 21478773 PMC4617830

[36] Gelenberg AJ. The prevalence and impact of depression. J Clin Psychiatry. 2010;71(3):e06 10.4088/JCP.8001tx17c 20331925

[37] Prince M, Bryce R, Albanese E, Wimo A, Ribeiro W, Ferri CP. The global prevalence of dementia: A systematic review and metaanalysis. Alzheimer's & Dementia: The Journal of the Alzheimer's Association. 2013;9(1):63–75.e2. 10.1016/j.jalz.2012.11.007 23305823

[38] Stinchcombe A, Kuran N, Powell S. Report summary. Seniors' Falls in Canada: Second Report: key highlights. Chronic diseases and injuries in Canada. 2014;34(2–3):171–4. 24991781

[39] Vaillancourt S, Shahin I, Aggarwal P, Pomedli S, Hayden L, Pus L, et al Using Archetypes to Design Services for High Users of Healthcare. HealthcarePapers. 2014;14(2):37–41. 25880862 10.12927/hcpap.2015.24107

[40] Thorpe KE, Ogden LL, Galactionova K. Chronic conditions account for rise in Medicare spending from 1987 to 2006. Health Aff (Millwood). 2010;29(4):718–24. 20167626 10.1377/hlthaff.2009.0474

[41] Zuvekas SH, Cohen JW. Prescription drugs and the changing concentration of health care expenditures. Health Aff (Millwood). 2007;26(1):249–57. 17211035 10.1377/hlthaff.26.1.249

