# Supplementary material for: Who Are the High-Cost Users? A Method for Person-Centred Attribution of Health Care Spending
Source: PLoS One. 2016 Mar 3;11(3):e0149179. doi: 10.1371/journal.pone.0149179 (PMC4777563; doi:10.1371/journal.pone.0149179)
Supplement: S1 Fig — (PDF) [file pone.0149179.s001.pdf]

S1 Figure. Main clinical groupings for person-centred episodes (PCE)

Reason for Health Care Use	Databases	Codes
1. Pregnancy	Canadian Institute for Health Information-Discharge Abstract Database (CIHI-DAD)	**Any most responsible diagnosis code of:**<br><br>Chapter XV: Pregnancy, childbirth and the puerperium<br>(O00-O99)<br>Z34, Z35<br>The codes included in this chapter are to be used for conditions related to or aggravated by the pregnancy, childbirth or by the puerperium (maternal causes or obstetric causes).
2. Low Birth Weight, Other Perinatal and Congenital Conditions	CIHI-DAD	**Any most responsible diagnosis code of:**<br><br>Chapter XVI: Certain conditions originating in the perinatal period (P00-P96) and congenital (Q00-Q99)<br>Includes conditions that have their origin in the perinatal period even though death or morbidity occurs later.
3. Post-admission events	CIHI-DAD	**All cases where with Type 2 in any diagnosis field**<br><br>**OR any diagnosis code in following**:<br>Y40-Y84,Y88, Y95
4. Trauma, Accidents, Injuries and Poisonings	National Ambulatory Reporting Systems (NACRS), CIHI-DAD	**Any diagnosis code of:**<br><br>S140, S1410, S1411, S1412, S1413, S1418, S1419, S240, S2410, S2411, S2412, S2413, S2418, S2419, S3400 S3410, S3411, S3412, S3413, S3418, S3419, S3430, S3438, T060, T061, V01–V99, W00–W99, X00–X99, Y00–Y36, Y85,Y86, Y89, Y90,Y91,Y96-Y98
5. Mental illness and addictions	CIHI-DAD, Ontario Mental Health Reporting System (OMHRS)	**Within CIHI-DAD, any most responsible diagnosis code of:**<br><br>F00-F99<br>G30, K70, R45, R46, R54<br>X60-X84, Y87<br><br>**All OMHRS admissions**

6. Ambulatory Care Sensitive Hospitalizations	CIHI-DAD	**For persons aged 0-74 and any most responsible diagnosis code** (based on the Manitoba Centre for Health Policy; http://mchp-appserv.cpe.umanitoba.ca):<br><br>Acute<br><br>• **Congenital Syphilis**<br>ICD-10-CA: A50<br>• **Imunization-Related:**<br>ICD-10-CA codes A35, A37, A80, I00, I01 (hemophilus meningitis for children ages 1-5 only, code G00.0)<br>• **Severe ENT Infections & otitis media:**<br>ICD-10-CA codes H66*, J02, J03, J06, J312 (*ICD-10-CA code H66 exclude CCI code 1.DF.53.JA-TS)<br>• Tuberculosis<br>ICD-10-CA codes A15--A19<br>• **Pneumonia:**<br>ICD-10-CA codes J13, J14, J15.3, J15.4, J15.7, J15.9, J16, J18 (patients with a secondary diagnosis of sickle-cell anemia, ICD-10-CA codes D57.0, D57.1, D57.2, D57.8, and patients less than two months of age are excluded)<br>• **Hypoglycemia:**<br>ICD-10-CA codes E16.0, E16.1, E16.2<br>• **Gastroenteritis:**<br>ICD-10-CA codes K52.2, K52.8, K52.9<br>• **Kidney/Urinary Infections:**<br>ICD-10-CA codes N10, N11, N12, N13.6, N15.1, N15.8, N15.9, N16.0-N16.5, N28.83-N28.85, N36.9, N39.0, N39.9<br>• **Dehydration/Volume Depletion:** ICD-10-CA code E86<br>• **Iron Deficiency Anemia:**<br>ICD-10-CA codes D50.1, D50.8, D50.9 (patients age 0-5 only)<br>• **Nutritional Deficiencies:**<br>ICD-10-CA codes E40-E43, E55.0, E64.3<br>• **Failure to Thrive:**<br>ICD-10-CA code R62 (patients less than one year of age only)<br>• **Pelvic Inflammatory Disease:**<br>ICD-10-CA codes N70, N73, N99.4 (female patients only, patients with a hysterectomy procedure coded are excluded, CCI codes 1.RM.87, 1.RM.89, 1.RM.91, 5.CA.89.CK, 5.CA.89.DA, 5.CA.89.GB, 5.CA.89.WJ, 5.CA.89.WK)<br>• **Dental Conditions:**<br>ICD-10-CA codes K02-K06, K08, K09.8, K09.9, K12, K13<br>• **Cellulitis:**<br>ICD-10-CA codes L03, L04, L08, L44.4, L88, L92.2, L98.0, L98.3

		(patients with any surgical procedure coded are excluded, except for incisions of skin and subcutaneous tissue, CCI codes 1.AX.53.LA-QK, 1.IS.53.HN-LF, I.IS.53.LA-LF, 1.JU.53.GP-LG, 1.KR.53.LA-LF, 1.OA.53.LA-QK, 1.SY.53.LA-QK, 1.YA.35.HA-W1, 1.YA.35.HA-X4, 1.YA.52.HA, 1.YA.52.LA, 1.YA.55.DA-TP, 1.YA.55.LA-TP, 1.YA.56.LA, 1.YB.52.HA, 1.YB.52.LA, 1.YB.55.DA-TP, 1.YB.55.LA-TP, 1.YB.56.LA, 1.YF.35.HA-W1, 1.YF.35.HA-X4, 1.YF.52.HA, 1.YF.55.DA-TP, 1.YF.55.LA-TP, 1.YF.56.LA, 1.YG.52.HA, 1.YG.52.LA, 1.YG.55.DA-TP, 1.YG.55.LA-TP, 1.YG.56.LA, 1.YR.52.HA, 1.YR.52.LA, 1.YR.56.LA, 1.YS.35.HA-W1, 1.YS.35.HA-X4, 1.YS.52.HA, 1.YS.52.LA, 1.YS.55.DA.TP, 1.YS.55.LA-TP, 1.YS.56.LA, 1.YT.35.HA-W1, 1.YT.35.HA-X4, 1.YT.52.HA, 1.YT.52.LA, 1.YT.55.DA-TP, 1.YT.55.LA-TP, 1.YT.56.LA, 1.YU.52.HA, 1.YU.52.LA, 1.YU.55.DA-TP, 1.YU.55.LA-TP, 1.YU.56.LA, 1.YV.35.HA-W1, 1.YV.35.HA-X4, 1.YV.52.HA, 1.YV.52.LA, 1.YV.55.DA-TP, 1.YV.55.LA-TP, 1.YV.56.LA, 1.YW.52.HA, 1.YW.52.LA, 1.YW.55.DA-TP, 1.YW.55.LA-TP, 1.YW.56.LA, 1.YX.52.HA, 1.YX.52.HA-AV, 1.YX.52.LA, 1.YX.56.LA, 1.YZ.35.HA-W1, 1.YZ.35.HA-X4, 1.YZ.52.HA, 1.YZ.52.LA, 1.YZ.55.DA-TP, 1.YZ.55.LA-TP, 1.YZ.56.LA)<br><br>Chronic<br><br>• **Grand mal status and other epileptic convulsions**<br>ICD-10-CA: G40, G41, R56<br>• **Chronic obstructive pulmonary diseases (COPD)**<br>Any most responsible diagnosis code of<br>ICD-10-CA: J41, J42, J43, J44, J47<br>Any most responsible diagnosis code of Acute lower respiratory infection, only when a secondary diagnosis of J44 in ICD-10-CA<br>ICD-10-CA: J10.0, J11.0, J12-J16, J18, J20, J21, J22<br><br>• **Asthma**<br>ICD-10-CA: J45<br>• **Diabetes**<br>ICD-10-CA: E10.0, E10.1, E10.6, E10.7, E10.9<br>E11.0, E11.1, E11.6, E14.7, E11.9<br>E13.0, E13.1, E13.6, E14.7, E13.9<br>E14.0, E14.1, E14.6, E14.7, E14.<br>• **Heart failure and pulmonary edema***<br>ICD-10-CA: I50, J81 *Excluding cases with cardiac procedures<br>• **Hypertension***<br>**ICD-10-CA:** I10.0, I10.1, I11<br>*Excluding cases with cardiac procedures<br>• **Angina***<br>**ICD-10-CA:** I20, I23.82, I24.0, I24.8, I24.9

		*Excluding cases with cardiac procedures<br>***List of cardiac procedure codes for exclusion:**<br>**CCI:** 1HA58, 1HA80, 1HA87, 1HB53, 1HB54, 1HB55, 1HB87, 1HD53, 1HD54, 1HD55, 1HH59, 1HH71, 1HJ76, 1HJ82, 1HM57, 1HM78, 1HM80, 1HN71, 1HN80, 1HN87, 1HP76, 1HP78, 1HP80, 1HP82, 1HP83, 1HP87, 1HR71, 1HR80, 1HR84, 1HR87, 1HS80, 1HS90, 1HT80, 1HT89, 1HT90, 1HU80, 1HU90, 1HV80, 1HV90, 1HW78, 1HW79, 1HX71, 1HX78, 1HX79, 1HX80, 1HX83, 1HX86, 1HX87, 1HY85, 1HZ53 rubric (except 1HZ53LAKP), 1HZ55 rubric (except 1HZ55LAKP), 1HZ56, 1HZ57, 1HZ59, 1HZ80, 1HZ85, 1HZ87, 1IF83, 1IJ50, 1IJ55, 1IJ57, 1IJ76, 1IJ86, 1IJ80, 1IK57, 1IK80, 1IK87, 1IN84, 1LA84, 1LC84, 1LD84, 1YY54LANJ
7. Cancer	CIHI-DAD	Any most responsible diagnosis code for Cancer (Included all “C” codes, no “D” cancer codes were included)<br><br>C00-C99
8. Acute planned surgical	CIHI-DAD. Same Day Surgery (SDS)	Acute planned admission for Surgery excluding cancer<br><br>Admit type = elective<br>Major Clinical Category (MCC) = surgery
9. Acute planned medical	CIHI-DAD	Acute planned admission for medical care excluding cancer<br><br>Admit type = elective<br>MCC = medical
10. Acute unplanned surgical	CIHI-DAD SDS	All other urgent surgical care admissions<br>Admit type = urgent code<br>MCC = surgical
11. Other acute unplanned medical	CIHI-DAD	All other urgent care medical<br>Admit type = urgent code<br>MCC = medical
12. Other hospitalized	CIHI-DAD SDS	All remaining CIHI DAD and SDS records fell into this category.
