# Supplementary material for: Who Are the High-Cost Users? A Method for Person-Centred Attribution of Health Care Spending
Source: PLoS One. 2016 Mar 3;11(3):e0149179. doi: 10.1371/journal.pone.0149179 (PMC4777563; doi:10.1371/journal.pone.0149179)
Supplement: S2 Fig — (PDF) [file pone.0149179.s002.pdf]

S2 Figure. Ten most prevalent conditions in each of the 12 defined person-centred categories

Categories	Codes	Description of code	Number (%) within the category
Pregnancy	**ICD-10**	***Data is captured from the Canadian Institute for Health Information-Discharge Abstract Database (CIHI-DAD). These codes reflect the Most Responsible Diagnosis.***	
	O68	Labour and delivery complicated by fetal stress [distress]	1,921(10.6)
	O34	Maternal care for known or suspected abnormality of pelvic organs	1,584 (8.8)
	O62	Abnormalities of forces of labour	1,139 (6.3)
	O60	Preterm delivery	1055 (5.8)
	O99	Other maternal diseases classifiable elsewhere but complicating pregnancy, childbirth and puerperium	1,032 (5.7)
	O42	Premature rupture of membranes	964 (5.3)
	O13	Gestational [pregnancy-induced] hypertension without significant proteinuria	908 (5.0)
	O32	Maternal care for known or suspected malpresentation of fetus	823 (4.5)
	O70	Perineal laceration during delivery	658 (3.6)
	O64	Obstructed labour due to malposition and malpresentation of fetus	650 (3.6)
Low Birth Weight, Other Perinatal and Congenital Conditions	**ICD-10**	***Data is captured in CIHI-DAD. These codes reflect the Most Responsible Diagnosis***	
	P07	Disorders related to short gestation and low birth weight, not elsewhere classified	4,806 (44.9)
	Z38	Liveborn infants according to place of birth	879 (8.2)
	P22	Respiratory distress of newborn	855 (8.0)
	P96	Other conditions originating in the perinatal period	219 (2.0)
	Q21	Congenital malformations of cardiac septa	212 (2.0)
	P28	Other respiratory conditions originating in the perinatal period	183 (1.7)
	P59	Neonatal jaundice from other and unspecified causes	146 (1.4)
	P05	Slow fetal growth and fetal malnutrition	121 (1.1)
	P24	Neonatal aspiration syndromes	113 (1.1)
	Q25	Congenital malformations of great arteries	109 (1.0)
Post-Admission Events	**ICD-10**	***Data is captured in CIHI-DAD. These codes reflect all cases with a Type 2 classification and/or any of the selected diagnosis codes of external causes of injury.***	
	T81	Complications of procedures, not elsewhere classified	8,459 (11.3)
	T84	Complications of internal orthopaedic prosthetic devices, implants and grafts	3,692 (4.9)
	N39*	Other disorders of urinary system	3,515 (4.7)
	E87*	Other disorders of fluid, electrolyte and acid-base balance	2,739 (3.6)
	T82	Complications of cardiac and vascular prosthetic devices, implants and grafts	2,705 (3.6)
	K91*	Post procedural disorders of digestive system, not elsewhere classified	2,364 (3.1)
	I48*	Atrial fibrillation and flutter	2,152 (2.9)
	T85	Complications of other internal prosthetic devices, implants and grafts	1,562 (2.1)
	J18*	Pneumonia, organism unspecified	1,534 (2.0)
	I95*	Hypotension	1,468 (2.0)

Trauma, Accidents, Injuries and Poisonings	**ICD-10**	***Data is captured from two sources, CIHI-DAD and the National Ambulatory Reporting System (NACRS). These codes reflect any of the diagnosis codes related to trauma, accidents, poisoning, or injuries.***	
	S72	Fracture of femur	10,210 (23)
	S82	Fracture of lower leg, including ankle	4,553 (10.3)
	S06	Intracranial injury	3,034 (6.8)
	S32	Fracture of lumbar spine and pelvis	3,009 (6.8)
	S42	Fracture of shoulder and upper arm	1,909 (4.3)
	X61	Intentional self-poisoning by and exposure to antiepileptic, sedative-hypnotic, antiparkinsonism and psychotropic drugs, not elsewhere classified	1,569 (3.5)
	S22	Fracture of rib(s), sternum and thoracic spine	1,547 (3.5)
	S52	Fracture of forearm	1,306 (2.9)
	S02	Fracture of skull and facial bones	769 (1.7)
	S09	Other and unspecified injuries of head	716 (1.6)
Mental Illness and Addictions	***ICD 10-DAD***	***Data is captured from three sources, CIHI-DAD, NACRS, and the Ontario Mental Health Reporting System (OHMRS). Within the CIHI-DAD and NACRS, codes reflect the most responsible diagnosis. All admissions were included for OHMRS.***	
	F05	Delirium, not induced by alcohol and other psychoactive substances	1,581 (13.3)
	F32	Depressive episode	1,401 (11.8)
	F10	Mental and behavioural disorders due to use of alcohol	1,297 (10.9)
	F03	Unspecified dementia	1,208 (10.2)
	F43	Reaction to severe stress, and adjustment disorders	870 (7.3)
	K70	Alcoholic liver disease	838 (7.1)
	G30	Alzheimer's disease	569 (4.8)
	F41	Other anxiety disorders	499 (4.2)
	F50	Eating disorders	277 (2.3)
	F29	Unspecified nonorganic psychosis	245 (2.1)
	**ICD9-OMHRS**		
	296	Manic depressive psychosis, involutional melancholia	9,997 (33.9)
	295	Schizophrenia	7,859 (26.7)
	missing	Missing	2,367 (8.0)
	298	Other psychoses	1,485 (5.0)
	300	Anxiety neurosis, hysteria, neurasthenia, obsessive compulsive neurosis, reactive depression	1,106 (3.8)
	292	Drug psychosis	1,066 3.6
	294	Amnestic syndrome	797 2.7
	309	Adjustment reaction	741 2.5
	303	Alcoholism	733 2.5
	304	Drug dependence, drug addiction	633 2.1

Ambulatory Care Sensitive Conditions		***Data is captured from CIHI-DAD. Codes reflect the most responsible diagnosis.***	
	J44	Other chronic obstructive pulmonary disease	6,976 (25.9)
	I50	Heart failure	3,779 (14)
	I20	Angina pectoris	2,386 (8.9)
	N39	Other disorders of urinary system	1,925 (7.2)
	E11	Type 2 Diabetes mellitus	1,631 (6.1)
	E10	Type 1 Diabetes mellitus	1,228 (4.6)
	G40	Epilepsy	1,218 (4.5)
	L03	Cellulitis	1,150 (4.3)
	J45	Asthma	968 (3.6)
	R56	Convulsions, not elsewhere classified	948 (3.5)
Cancer		***Data is captured from CIHI-DAD and Same Day Surgery (SDS). Codes reflect the most responsible diagnosis.***	
	C50	Malignant neoplasm of breast	7,806 (15.1)
	C67	Malignant neoplasm of bladder	4,880 (9.4)
	C61	Malignant neoplasm of prostate	4,278 (8.3)
	C18	Malignant neoplasm of colon	4,234 (8.2)
	C34	Malignant neoplasm of bronchus and lung	4,123 (8)
	C78	Secondary malignant neoplasm of respiratory and digestive organs	2,323 (4.5)
	C44	Other malignant neoplasms of skin	2,056 (4)
	C79	Secondary malignant neoplasm of other sites	2,048 (4)
	C20	Malignant neoplasm of rectum	1,889 (3.6)
	C73	Malignant neoplasm of thyroid gland	1,752 (3.4)
Acute Planned Surgical		***Data is captured from CIHI-DAD and Same Day Surgery (SDS). Codes reflect the all planned surgeries, excluding cancer.***	
	M17	Gonarthrosis [arthrosis of knee]	17,137 (7.0)
	H26	Other cataract	16,754 (6.8)
	I25	Chronic ischaemic heart disease	10,816 (4.4)
	D12	Benign neoplasm of colon, rectum, anus and anal canal	8,748 (3.6)
	M16	Coxarthrosis [arthrosis of hip]	8,585 (3.5)
	Z08	Follow-up examination after treatment for malignant neoplasm	7,660 (3.1)
	H25	Senile cataract	5,741 (2.3)
	Z12	Special screening examination for neoplasms	4,850 (2)
	N40	Hyperplasia of prostate	4,799 (2)
	Z09	Follow-up examination after treatment for conditions other than malignant neoplasms	4,466 (1.8)
Acute Planned Medical		***Data is captured from CIHI-DAD. Codes reflect the major clinical category, excluding cancer.***	
	Z53	Persons encountering health services for specific procedures, not carried out	2,141 (27.5)
	Z51	Other medical care	1,197 (15.4)
	I25	Chronic ischaemic heart disease	388 (5)
	Z54	Convalescence	220 (2.8)
	Z75	Problems related to medical facilities and other health care	126 (1.6)

	J18	Pneumonia, organism unspecified	96 (1.2)
	I21	Acute myocardial infarction	94 (1.2)
	E84	Cystic fibrosis	88 (1.1)
	I48	Atrial fibrillation and flutter	87 (1.1)
	Z50	Care involving use of rehabilitation procedures	85 (1.1)
Acute Unplanned Surgical		***Data is captured from CIHI-DAD. Codes reflect the major clinical category.***	
	I21	Acute myocardial infarction	5,502 (19.3)
	K35	Acute appendicitis	2,327 (8.1)
	K80	Cholelithiasis	2,281 (8.0)
	K56	Paralytic ileus and intestinal obstruction without hernia	1,019 (3.6)
	I25	Chronic ischaemic heart disease	1,010 (3.5)
	I44	Atrioventricular and left bundle-branch block	889 (3.1)
	I20	Angina pectoris	649 (2.3)
	N20	Calculus of kidney and ureter	597 (2.1)
	N13	Obstructive and reflux uropathy	547 (1.9)
	K85	Acute pancreatitis	470 (1.6)
Acute Unplanned Medical		***Data is captured from CIHI-DAD. Codes reflect the major clinical category.***	
	I21	Acute myocardial infarction	8,741 (6)
	J18	Pneumonia, organism unspecified	7,816 (5.4)
	I50	Heart failure	6,867 (4.7)
	J44	Other chronic obstructive pulmonary disease	6,825 (4.7)
	I63	Cerebral infarction	5,200 (3.6)
	Z51	Other medical care	5,140 (3.5)
	R07	Pain in throat and chest	4,323 (3.0)
	I48	Atrial fibrillation and flutter	4,094 (2.8)
	K56	Paralytic ileus and intestinal obstruction without hernia	3,347 (2.3)
	N39	Other disorders of urinary system	2,951 (2.0)
